# Microbiota-derived small molecule genotoxins: host interactions and ecological impact in the gut ecosystem

**DOI:** 10.1080/19490976.2024.2430423

**Published:** 2024-11-18

**Authors:** Ellen L. Zechner, Sabine Kienesberger

**Affiliations:** a Institute of Molecular Biosciences, University of Graz, Graz, Austria; b BioTechMed-Graz, Graz, Austria; c Field of Excellence BioHealth, University of Graz, Graz, Austria

**Keywords:** Bacterial antagonism, DNA damage, secondary metabolites, colorectal cancer, inflammatory bowel disease, colibactin, tilimycin, tilivalline, indolimine, competitive interference, colonization resistance, Klebsiella, early-life colonization, colitis, necrotizing enterocolitis

## Abstract

The human intestinal tract is densely colonized by a microbial community that is subject to intense competition. Bacteria in this complex habitat seek to outcompete their neighbors for nutrients and eliminate competitors with antibacterial toxins. Antagonism can be mediated by diverse effectors including toxic proteins and small molecule inhibitors that are released extracellularly or delivered by specialized secretion systems to targeted cells. Two prototypical microbiota-derived enterotoxins, colibactin and tilimycin, and the newly discovered family of indolimines represent an expanding group of non-proteinaceous small molecules which specifically target DNA. In addition to cell killing, they generate mutations and genome instability in intoxicated microbes and host cells alike. They have been studied in detail because of their direct toxicity to human cells and important etiological roles in intestinal pathologies. Increasing evidence, however, reveals that these commensal genotoxins are also mediators of interbacterial antagonism, which impacts gut microbial ecology. In this review, we illustrate the functional versatility of commensal genotoxins in the gut ecosystem.

## Introduction

Genotoxins are agents or chemicals that cause DNA or chromosomal damage, thereby causing mutations. The discovery of colibactin in 2006^1^ verified that the vast metabolic potential of the human gut microbiota also encompasses synthesis of small molecules with the capacity to damage host cellular DNA and drive genetic instability. Research following the discovery of colibactin identified another DNA-alkylating secondary metabolite, tilimycin, which targets cells of the host.^2–5^ Awareness that colibactin-producing commensal strains of *Escherichia coli* and tilimycin-secreting species of *Klebsiella* are among the first colonizers of the infant gut and indeed may persist in the host throughout life, spurred extensive investigation of the cytopathic activities of colibactin and tilimycin in mammalian systems. A recent systematic functional screen aiming to detect residents of the colon microbiota which secrete DNA-damaging small molecules (<3 kDa) identified numerous toxigenic strains harbored by patients with inflammatory bowel diseases.^6^ Cao and coauthors^6^ then focused on the facultative anaerobic enteric bacterium *Morganella morganii* to identify and characterize the family of genotoxic indolimines. Their demonstration that indolimine synthesis in the murine intestine increased tumor burden underscored the expanding role of bacterial genotoxic metabolites in colorectal carcinogenesis.^7^ One clear implication of these discoveries is that the gut ecosystem represents a rich reservoir of diverse and potentially abundant pro-mutagenic natural products with oncogenic potential.

The importance of the microbiota in the development of colorectal cancer (CRC) has been the topic of intensive research.^8–13^ Much progress has been made toward understanding the role prototypical commensal genotoxins play in intestinal injury and the earliest stages of tumorigenesis. Particularly, several excellent reviews have described advances in colibactin research including its biosynthesis, structure-activity relationships, and others focusing on its role in carcinogenesis.^14–18^ In this review, we widen the scope to include functions of the *Klebsiella* metabolites and the newly discovered family of indolimines to highlight both the shared and unique features of these paradigms. Moreover, while detailed knowledge of the mechanisms underlying their association to disease continues to emerge, the ecological impacts of intestinal genotoxicity are less understood. In this intensively competitive environment these systems add to the armory of antibiotics, bacteriocins, toxins, and delivery systems bacteria have evolved to engage in interspecies conflict. For detailed descriptions of these diverse antagonistic mechanisms and the protective responses, they induce, we refer readers to several comprehensive reviews.^19–24^ Here, we summarize the current knowledge of the aggressive phenotypes mediated by commensal genotoxins, their capacity to modulate bacterial behaviors, promote mutation and ultimately alter the course of evolution in the gut ecosystem.

## Prevalence of toxigenic commensal species

**Figure 1.**
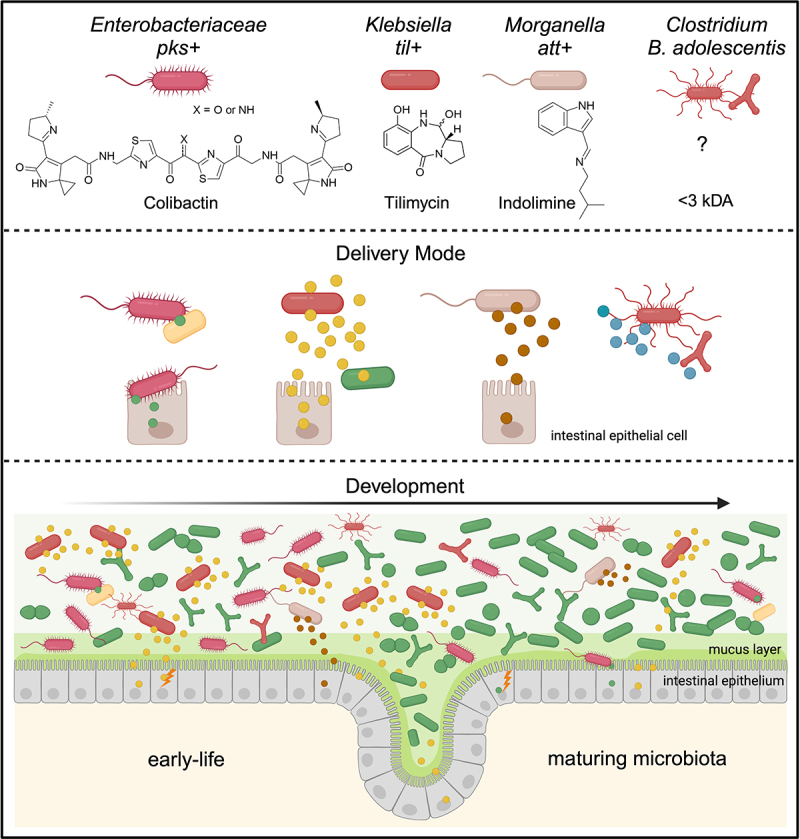
Bioavailability of small molecule genotoxins in the gut ecosystem. Bacteria harboring biosynthesis genes for the DNA-damaging substances shown. The proposed active species of colibactin shown here is chemically unstable and delivered to targeted host and bacterial cells by a contact-dependent mechanism. The stable, bioactive genotoxins of *Klebsiella*, *Morganella, Clostridium* and *Bifidiobacterium adolescentis* are secreted independently of cell contact and freely diffusible. The neonate gut can be seeded by genotoxic commensal species from maternal and environmental sources. They persist at lower abundance during its maturation and have potential to drive genetic instability over the lifespan. Created in BioRender. Kienesberger, S. (2024) BioRender.com/r95n215.

The small molecule genotoxin colibactin (Figure 1) can enter the human gut ecosystem very early in the life of a newborn.^25,26^ It is produced by several commensal and pathogenic *Enterobacteriaceae*, including *Escherichia coli* belonging to phylogenetic group B2, *Klebsiella pneumoniae*, *Klebsiella aerogenes*, and *Citrobacter koseri*.^27^ Mother to infant transmission of colibactin-positive *E. coli* has been reported.^28^ The biosynthetic machinery necessary for the production and transport of the hybrid non-ribosomal peptide-polyketide toxin is encoded by 19 genes (*clbA-clbS*) located on a 54-kb genomic island called alternatively *clb* or *pks*.^1^ Prevalence of the colibactin biosynthetic gene cluster (BGC) in commensal B2 strains of *E. coli* was 34% in a study investigating fecal isolates from healthy individuals.^1^ Strains belonging to this phylogenetic group have a superior capacity to colonize infants compared to other groups^26^ and their long-term persistence in the colon is associated with carriage of the *pks* locus.^29,30^ The incidence of B2 strains in high-income countries continues to rise.^31,32^ It is currently estimated that potentially genotoxic *pks*^+^ *E. coli* strains are present in the microbiomes of 10–30% of people in the Western world.^33–35^ By comparison, the detection rate in CRC patients is > 60%.^34,36^ The *pks* genomic island spreads in enterobacteria by horizontal gene transfer. *K. pneumoniae* is a common opportunistic pathogen in hospitals that can cause a variety of serious, drug-resistant infections.^37^ The *pks* locus is mobilized within the species *K. pneumoniae* by integrative and conjugative elements.^38^ A recent large-scale analysis of *K. pneumoniae* genomes found that 9% (245/2709) are *pks*+. Notably, the majority of these (142; 58%) belong to sequence type (ST)23, which is also the main sequence type of hypervirulent *K. pneumoniae*.^39^

The *til* island harbors two operons of biosynthesis genes which are required to generate the genotoxic non-ribosomal peptide tilimycin, a separate transcriptional unit of self-immunity genes and a gene for transcriptional regulation.^2,40,41^ The final enzymatic product of the *til* biosynthetic pathway undergoes ring closure to form the genotoxin tilimycin.^2^ *til+ Klebsiella* spp are indole producers and in the gut ecosystem a fraction of the available tilimycin reacts spontaneously with endogenous and microbiota-derived indole to form a second cytotoxic peptide, tilivalline.^2,3^ Tilivalline lacks DNA-binding activity but exhibits a unique binding interaction with tubulin that stabilizes polymerized microtubules blocking mitosis.^4^ The resulting apoptosis compromises gut barrier integrity.^40^ Functional *til* genes are required for *Klebsiella* to cause antibiotic-associated hemorrhagic colitis (AAHC).^4,40^ Gut residents carrying the *til* locus belong exclusively to the *Klebsiella oxytoca* species complex (KoSC). Similar to *pks*+ bacteria, toxigenic species of this complex (*K. oxytoca, K. michiganensis, K. grimontii* and *K. pasteurii*) colonize infants during *de novo* assembly of the gut microbiota.^5,42–44^ Colonization by *Klebsiella* also follows premature human births.^45–48^ Prevalence of the KoSC during the first weeks of life range from 20% to 70% in high-income countries.^5,42,44,49,50^ The relative abundance of the KoSC in the postnatal intestine can be very high but decreases with microbiome maturation in the first years of life (Figure 1).^5,44^ It is currently estimated that ca. 50% of human fecal isolates of KoSC secrete *til* metabolites.^50,51^

The aspartate aminotransferase (*aat*) gene of *Morganella morganii* is essential for synthesis of the indolimine family of small molecule genotoxins.^6^ Cao et al.^6^ reported broad conservation of this gene across *M. morganii* genomes implying that indolimine production may be common in this species. *M. morganii* can be acquired by mother-infant transmission^52^ and are present in 2% to 3% of infants in the first weeks of life.^44^ Similar to colonic mucosa-associated *pks+ E. coli* this bacterium is prevalent in inflammatory bowel disease (IBD)^53^ patients and enriched in cancerous tissues compared with controls.^36,54^ IBDs, comprising mainly ulcerative colitis and Crohn’s disease, are complex disorders. An increasing body of evidence suggests that microbiota-derived small molecules play a role in the etiology of IBD. To gain insights into the molecular and functional details underlying these associations, the Donia laboratory performed a multi-cohort analysis of small molecule BGCs in metagenomic samples of the gut microbiome from IBD patients.^55^ They found numerous small molecule BGCs significantly enriched in the IBD microbiome compared to those of healthy controls including, strikingly, both the *pks* and *til* genotoxicity loci.^55^ This powerful and generalizable approach adds compelling evidence that commensal genotoxins are molecular mediators of microbiome-associated diseases, including, specifically, IBD.

## Delivery mode, stability, biogeography

The bioavailability of small molecule genotoxins in the gut ecosystem differs substantially (Figure 1). The indolimines and tilimycin are diffusible agents, which enables their activities to be dispersed over distances. Tilimycin is secreted into the intestinal lumen in a stable, bioactive form and is therefore able to communicate with the host epithelium and gut microbes independently of physical contact. Pöltl et al.^5^ charted the load of genotoxic bacteria and the longitudinal distribution of luminal tilimycin in the intestines of antibiotic-treated mice colonized with *til+ K. oxytoca*. Producing- organisms and the *til* metabolites were present in all compartments and particularly concentrated in cecum and colon. Pöltl’s study also showed that despite the protective measures shielding the colonic stem cell niche from luminal contents – anatomical restriction, secreted mucus, effector absorption by colonocytes and microbes within the crypts – tilimycin permeated the depths of the crypts to drive emergence of colorectal stem cell mutations (Figure 1). Histological examination revealed no abnormalities in the colon in this model, thus the somatic variation occurred in structurally intact crypts. This differentiates the efficacy of tilimycin from other enterotoxins such the genotoxic protein UshA produced by attaching/effacing enteropathogenic *E. coli* and the *C. difficile* toxin TcdB which promote colonic stem cell dysfunction once other microbial features disrupt intestinal cellular organization.^56,57^ Whether other features of the KoSC contribute to the mutational environment is currently unknown. The biogeography of indolimine-producers in the intestine is likewise yet to be explored.

Unlike the indolimines and tilimycin, colibactin is chemically highly unstable. Metabolomics are insufficient for its quantification; thus, concentrations of structural precursors such as the prodrug motif *N-*myristoyl-D-asparagine are monitored as a surrogate. Moreover, colibactin is transmitted from producing bacteria to recipient cells by a cell contact-dependent mechanism which restricts its spatial range to those cells within close physical proximity (Figure 1).^1,58,59^ The requirement for cell-to-cell delivery creates additional barriers to effective interaction with the host. For example, colibactin-mediated activities *in vitro* and in mouse models are attenuated by an adherent mucous layer on epithelial cells.^60,61^ Despite this protective shield, colibactin-dependent damage is observed along the entire length of the gastrointestinal tract in animal models of infection.^25^ Consistent with the need for contact-induced delivery, colibactin-producing organisms may also apply microbe-host signaling strategies to stimulate increased accessibility of the host epithelium. Raisch et al.^62^ showed that adherent-invasive and colon cancer-associated strains of *E. coli* induce carcinoembryonic antigen-related cell adhesion molecule 6 (CEACAM6) expression, a receptor involved in adhesion of pathogenic *E. coli*. These *pks*+ bacteria were able to persist and promote low-grade inflammation and cell proliferation in a chronic infection model of CEACAM6-expressing mice. Moreover, a defective mucosal barrier is characteristic of IBD as well as neoplastic tissue and in mucosa-associated communities degradation of protective mucus by co-occurring bacteria such as *Bacteroides fragilis* can promote access of *E. coli* to host epithelial cells, increasing delivery of colibactin.^34^

## Metabolite-host interactions

**Figure 2.**
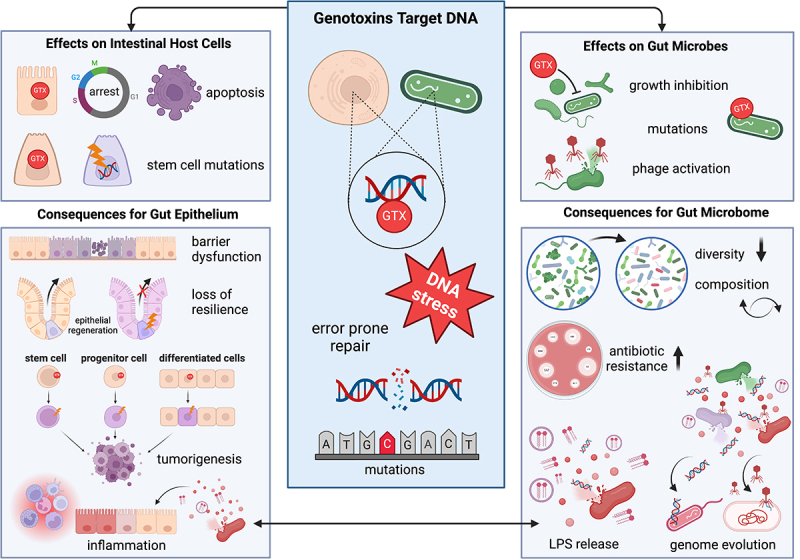
Cytopathic activities of commensal genotoxins (GTX) broadly impact the gut ecosystem. Genotoxic bacteria induce DNA damage, activate stress response and DNA repair pathways, promote mutations and genomic instability (center). Host exposure (left panel) influences cell cycle dynamics, drives somatic genetic variation and alters patterns of apoptosis, proliferation, and differentiation. Consequences include loss of epithelial barrier function, diminished regenerative capacity, mucosal inflammation, and tumorigenesis. DNA stress in microbes alerts damage-signaling cascades and defenses (right panel). Genotoxicity promotes mutation, phage activation and cell death ultimately driving genome evolution, reduced diversity, altered community structure and function and release of inflammatory stimulants such as fimbriae and lipopolysaccharide (LPS). Created in BioRender. Kienesberger, S. (2024) BioRender.com/q98u631.

Colibactin alkylates deoxyadenosines within AT-rich motifs resulting in inter-strand cross-links (ICLs) in infected cells.^63–66^ ICLs impede DNA replication and require repair in a replication-coupled manner.^67^ The damage causes genomic instability by induction of double-strand breaks in DNA (Figure 2).^68^ When the load of toxigenic bacteria is high, the cell cycle arrests irreversibly and apoptosis follows.^1,63,68^ At lower densities, which better reflect the abundance of commensal producers, cells repair most but not all damage faithfully. Errors in repair generate chronic mitotic and chromosomal aberrations together with increased frequency of gene mutation arising apparently by translesion synthesis past the colibactin-ICL remnant.^67^ A study using neonatal rat pups born to mothers fed commensal *pks*+ *E. coli* revealed increased DNA double-strand breaks in gut epithelial cells during the first week of life compared with pups originating from mothers treated with a non-producing *E. coli clbA*- mutant.^25^ Persistence of *pks+* bacteria into adulthood in this model was associated with perturbed intestinal homeostasis including altered patterns of apoptosis, proliferation, and differentiation, and increased gut permeability.

DNA alkylation (Figure 2) by the tricyclic pyrrolobenzodiazepine (PBD) tilimycin occurs in human cultured cells and enterocytes of mice colonized with *til+ K. oxytoca*.^4,69^ Adduct formation^69^ activates the DNA damage response and repair is mediated particularly by the transcription-coupled nucleotide excision repair pathway.^4^ The mutations induced in repair are predominantly single base substitutions (SBS) in guanines flanked by purines (Pu-G-Pu),^5^ consistent with the preferred-binding sites of canonical PBDs produced by *Streptomyces* spp.^70^ Insertions and deletions were also observed in marker genes of cultured human cells.^5^ Notably, the genetic variation detected in these studies arose from exposure to physiologically relevant,^71^ low- and sub-micromolar concentrations of tilimycin.^5^ The interactions of tilimycin and tilivalline with their distinct molecular targets independently cause cell cycle arrest. The resulting apoptosis induced in intestinal epithelial cells causes loss of barrier integrity and provides the etiologic mechanism underlying AAHC in children and adults.^4,40,72,73^ Moreover, toxigenic KoSC members have been associated with necrotizing enterocolitis (NEC), the most common and lethal inflammatory bowel disease in infants.^46,48,74,75^

An indolimine-secreting *M. morganii* isolate expressing active aspartate aminotransferase but not an *aat* deficient mutant damaged DNA substrates in cell-free assays.^6^ Exposure of cultured Hela cells to indolimines induced formation of phosphorylated histone H2AX (γH2AX) foci, a sensitive biochemical marker for DNA double-strand breaks.^6^

## Oncogenic potential

Colorectal cancers exhibit substantially increased mutational burdens relative to normal cells. By repeatedly provoking low-grade DNA damage at the enterocyte level commensal genotoxic metabolites may thus promote successive somatic genetic change leading to CRC (Figure 2). To date, however, the role played by the timing and duration of commensal genotoxin exposure in determining CRC risk is poorly understood.^76^ Pöltl et al.^5^ recapitulated in conventional C57BL/6J mice the transient expansion of genotoxic *Klebsiella* observed in patients during antibiotic therapy.^72,77^ In this model, exposure of the intestinal epithelium to tilimycin over a timescale of days was sufficient to drive heightened somatic variation in the colorectal stem cell compartment which became stabilized into wholly mutant colonic crypts. Brief exposure to *pks+ E. coli* was also sufficient to cause mutations that lead to changes in growth factor dependence and differentiation in cell culture models.^78^ Preclinical studies using genetically susceptible mouse models demonstrated that common *pks*^*+*^ *E. coli* strains increased tumor burden compared to non-producing control strains.^34,36,79,80^ Similarly, compared with an *aat* mutant, indolimine-producing *M. morganii* increased gut permeability and exacerbated colon tumor burden in gnotobiotic mice in the context of a mock community of human gut microbes.^6^

Comparison of the prevalence or abundance of *Morganella* in primary gastrointestinal (GI) tumors compared to non-GI tumors identified increased levels of this genus in adenocarcinomas of the colon, rectum and stomach.^6^ Abnormal colonization of colonic adenomas, carcinomas and mucosa of patients with colon cancer is also a feature of adherent *E. coli* and *pks+* strains.^36,81–83^

Strong evidence supporting a direct etiologic role for colibactin in CRC was recently provided by two laboratories who independently identified the mutational outcomes of colibactin exposure.^84,85^ The observed mutational signatures – single base substitutions (SBS) by thymine and deletion of a specific 5-base DNA motif that consists predominantly of adenine – fits well with colibactin’s capacity to alkylate adenines on opposing strands of DNA. The mutational signatures, SBS-*pks* (SBS88) and an indel signature, ID-*pks* (ID18), were then identified in human tumors, notably in the *APC* (adenomatous polyposis coli) gene and numerous known CRC driver mutations. Lineage analyses suggest that these co-occurring *pks*-mutations arose during early childhood.^86^

## Metabolite – microbe interactions

The direct DNA-damaging activity of this group of bacterial metabolites supported the hypothesis that their presence in the gut could have powerful effects on the microbial community (Figure 2). Further, it stands to reason that antagonism of bacterial competitors represents their true evolutionary purpose. Microbiota composition is governed by two types of competition: exploitative competition, which occurs indirectly through resource consumption, and interference competition, whereby one individual directly harms another.^87^ Commensal genotoxins antagonize other bacteria directly; thus, in an ecological context they are important for interference competition. It is intuitive that many factors will govern how effectively a genotoxic metabolite shapes the enteric microbial community. Determinants of their efficacy include the size and distribution of the producing population, mode of toxin delivery, and the quantity, stability and spatial range of secreted genotoxins. The latter properties are relevant for the diffusible indolimines and tilimycin.

Uptake of the substances by sensitive bacteria should induce DNA stress, drive genetic variation, and inhibit growth of recipient cells (Figure 2). In which case, the impact of the toxigenic environment on both community structure and its functional interactions *in vivo* would be predicted to have direct and indirect consequences for microbial evolution.^88^ Kienesberger et al.^71^ treated fecal bacteria from healthy human donors with physiologically relevant quantities of tilimycin, i.e., quantities present in AAHC patient samples. They demonstrated that this peptide has antibacterial activity across multiple phyla including *Firmicutes* and *Bacteroidetes*, which constitute the majority of the intestinal microbiota. Transient synthesis of tilimycin in the murine gut (driven by antibiotic-induced dysbiosis) antagonized niche competitors, reduced microbial richness and altered taxonomic composition of the microbiota both during and following exposure. Pronounced shifts among Bacteroidales species were observed; a bacterial order which comprises some of the most abundant Gram-negative bacteria of the human gut microbiota. The large family of *Enterobacteriaceae* that includes early-life colonizers of the human gut was also particularly sensitive. This study further showed that tilimycin secretion *in vivo* increased rates of mutagenesis in co-resident opportunistic pathogens such as *K. pneumoniae* and *E. coli*, driving *de novo* emergence of antibiotic resistance.^71^ Tilimycin is thus predicted to have broad spectrum pro-mutagenic activity in surviving gut microbes and thereby generally expand the reservoir of antibiotic resistance in colonized individuals.

Colibactin is deployed by a contact-dependent system, thus it cannot kill cells at a distance but may be very effective at small length scales. Precedence is provided by mixed species wound infections where a uropathogenic strain of *E*. *coli* was shown to use toxin delivery to antagonize the growth and survival of Staphylococci.^89^ In this model, colibactin killed *Staphylococcus aureus* by causing irreparable DNA damage. The antibacterial activities of colibactin are believed to benefit producers in establishing and expanding their niche in the human gut ecosystem. This function may be particularly important during the initial colonization of infants.^22^ Tronnet et al.^90^ measured the effect of colibactin-producing microbes on *de novo* assembly of the gut microbiome in murine neonates following mother-to-pup transmission of a *pks+* extraintestinal pathogenic strain of *E. coli* or its non-genotoxic mutant. They observed colibactin-dependent phylum-level changes and a reduction in overall abundance within the first weeks of life. A mouse isolate of *pks+ E. coli* depleted *B. fragilis* and *Enterococcus* spp in the adult murine intestine and colibactin appears to specifically target *B. fragilis* in human carriers.^54^

An alternative mechanism of antagonism was described by Silpe et al.^59^ who showed under laboratory conditions that colibactin producers could affect targeted bacteria – including *E. coli*, *Salmonella, Staphylococcus, Citrobacter*, and *Enterococcus* – when the canonical SOS response induced by DNA damage activates resident prophages. These authors propose that induction of temperate bacteriophage by colibactin-producing bacteria is a broad-spectrum activity affecting phylogenetically distinct Gram-negative and Gram-positive bacteria. Accordingly, phage activation and the resulting cell lysis in the context of complex microbial communities are hypothesized to shape gut microbiota composition and function via a mechanism distinct from general growth inhibition. Moreover, this form of interbacterial antagonism could drive genome evolution in general as the DNA released from killed cells can be taken up and integrated into other bacterial genomes (Figure 2). The sum of these findings suggests that also in human hosts, the antibacterial activities of small molecule genotoxins have broad ecological impacts.

## Colonization resistance

**Figure 3.**
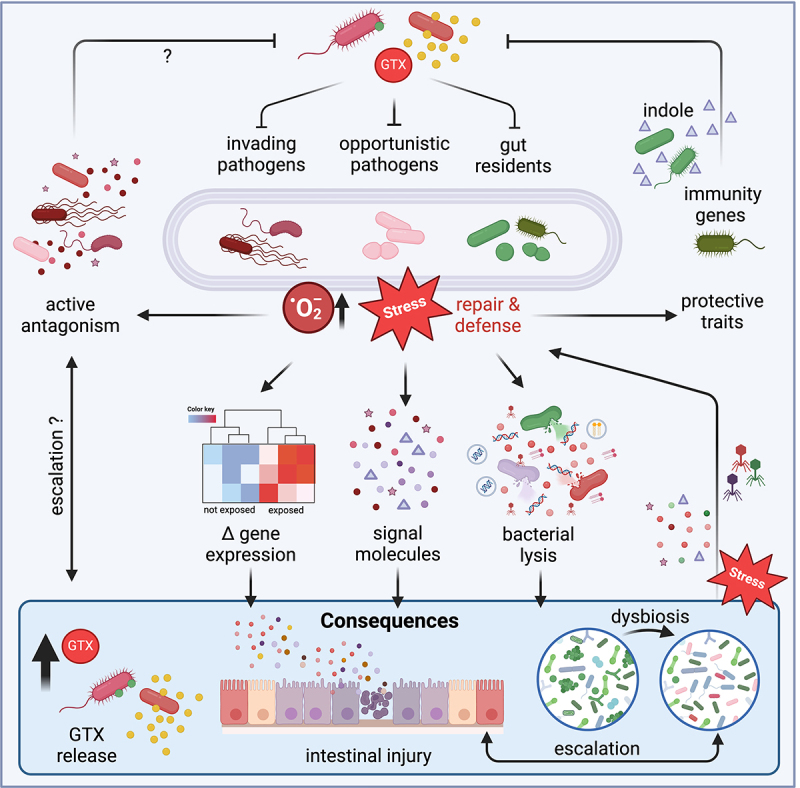
Small molecule genotoxins mediate bacterial antagonism. Colibactin inhibits multiple bacterial species and tilimycin exerts cross-phyla antibiotic activity. When directed against pathogens toxicity contributes to colonization resistance. In targeted bacteria genotoxins incite stress responses. Adaptive protective traits include carriage of orphan immunity genes in non-producers and indole signaling to downregulate tilimycin production. Cellular damage in targeted cells activates repair and presumably in some cases defense pathways. Whether complex response pathways enable competitors to attack genotoxic bacteria preemptively or respond aggressively to direct assault remains an open question (??). If so, then escalation of interbacterial warfare involving the genotoxic foe or nontoxic bystanders, exacerbated genotoxicity, general amplification of danger signaling, increased rates of horizontal gene transfer and cell lysis are all conceivable outcomes causing in turn broader microbiota depletion and intestinal injury. Created in BioRender. Kienesberger, S. (2024) BioRender.com/w24v345.

Commensal microbiota protect both hosts and the established community from colonization and infection by invading pathogens.^91^ Release of microbiota-derived antimicrobial toxins is one strategy contributing to ecosystem defense. The discovery of bactericidal activity for commensal genotoxins implied that these metabolites would not only drive interference competition in gut communities but also contribute to colonization resistance (Figure 3). Because genotoxic metabolites specifically target DNA the ensuing genetic instability can mediate resistance to enteric pathogens by multiple mechanisms including direct cell killing, pro-mutagenic activity, or prophage induction and cell lysis. Indirectly, as noted above, commensal genotoxins alter the species composition generally and thus the physical and chemical environment encountered by pathogenic bacteria in the gastrointestinal ecosystem.^88^ The resulting structural and biochemical disruption can alter niche occupation or shift the abundance of luminal resources, metabolic end products and molecules that modulate oxygen levels as well as host immunity.^91,92^ As an example of such toxin-induced indirect effects, infection studies using insect larvae revealed that host cells that encounter colibactin increase secretion of antimicrobial factors.^58^

Murine infection models were used to demonstrate genotoxin-mediated protection of the host from incoming pathogens. Chen et al.^58^ showed that colibactin produced by murine isolates of *E. coli* kill *Vibrio* spp. very effectively enabling these organisms to antagonize the important diarrheal pathogen *V. cholerae* during mouse colonization. The bactericidal effect is caused by intracellular accumulation of reactive oxygen species and DNA damage. Multiple serotypes of *V. cholerae* including the O1 and O139 serogroups responsible for epidemic disease are colibactin sensitive. Consistent with this finding, a further analysis of human metagenomic data from Bangladesh^93^ revealed that the presence of colibactin-producing organisms is associated with disease outcomes.^58^

Tilimycin is broadly effective against the *Enterobacteriaceae* ,^71^ and this family includes commensal species as well as intestinal pathogens such as *Salmonella*, *Shigella*, *Yersinia*, *Citrobacter*, and enteropathogenic *E. coli*. Endogenous communities of gut bacteria harboring KoSC members or pre-colonization of mice with single KoSC strains protected mice from infection with *Salmonella enterica* serovar (*S*.) Typhimurium, *K. pneumoniae* and *E. coli* .^94–96^ Competition over mono- and polysaccharide use contributes to the underlying mechanisms,^94–96^ and – as shown for *S*. Typhimurium – tilimycin-mediated bactericidal activity plays a significant additional role in attenuating colonization and virulence.^97^ The ability of KoSC bacteria to reduce *S*. Typhimurium fitness is broadly conserved in human fecal isolates, particularly among *K. grimontii* and *K. oxytoca* species. These few examples illustrate the ecological benefit conferred by genotoxin-producing members of the gut community by restricting ecosystem susceptibility to pathogens.

## Microbial countermeasures to genotoxicity

Like most antibacterial toxin systems, the *til* and *pks* islands carry self-immunity genes. Their products function intracellularly to limit damage to the genomes of producing cells. Examples include late-stage activation of structural intermediates in the periplasmic space (ClbP), deactivating modifications of the genotoxin (ClbS), increasing efflux from *til+ Klebsiella* (MfsX) or by expanding the repertoire of DNA repair pathways available to tilimycin producers with a dedicated repair enzyme (UvrX).^71,98–100^ Similarly, confrontations with genotoxins select for emergence of protective traits in susceptible bacteria. Acquisition of orphan immunity genes to neutralize small molecule antimicrobials not made by the organisms themselves may illustrate a general adaptation allowing diverse microbial communities to subvert antagonism (Figure 3).^20,101^ Expression of such a resistance determinant has been shown to protect a heterologous host from the genotoxicity of the cognate metabolite.^59,71,89^ Indeed, functional ClbS-like proteins are encoded by a wide range of unrelated bacteria that lack the *pks* locus. Maintenance of orphan *clbS* genes may thus represent an effective adaptation by prophage-carrying bacteria to limit their exposure to colibactin from neighboring cells allowing in turn more stable co-existence. This finding is consistent with analyses of other mature gut communities. Orphan immunity genes such as those belonging to the acquired interbacterial defense (AID) and recombination-associated AID systems of *Bateroidales* are widespread in adult gut metagenomes. These loci are thought to acquire genes to serve an adaptive immune function for defense against toxins of antagonizing strains.^102^ Moreover, comparison of the capacities for antagonism and resistance in complex soil communities harboring *Streptomyces* spp. reveal that the ability to resist antagonism by small molecule antimicrobials was a much more widespread trait than the ability to actively inhibit another strain.^101^

Indole and its derivates are involved in intraspecies, interspecies, and interkingdom signaling in microbial ecosystems.^103^ The capacity to produce indole from tryptophan by tryptophanase is ubiquitous among gut bacteria, particularly *Bacteroides* spp.^104^ Variation in indole availability alters expression of virulence factors in several gut pathogens^105–108^ and indole production was similarly shown to lower tilimycin secretion in the murine intestine.^109^ Bacterial indole lowers levels of the *til* biosynthesis gene transcripts *npsA* and *npsB*.^109^ Indole also enhances conversion of tilimycin to tilivalline, which is a strong agonist of the host pregnane X receptor. In response, this master regulator activates detoxifying genes.^109^ Microbiota-derived indole is thus an effective countermeasure to the enterotoxicity of *til*+ bacteria (Figure 3). Similarly, microbiota-derived indolimines are direct ligands for another xenobiotic receptor expressed in the intestinal tract, the aryl hydrocarbon receptor (AHR).^110^ Activation of this environmental sensor-induced transcription of AHR target genes to promote metabolism of xenobiotics and enhanced *IL6* expression in combination with cytokine treatment in cultured human cells. These responses may support AHR-mediated maintenance of intestinal homeostasis in the presence of indolimine-producing bacteria.

## Contextual factors alter genotoxin production

Research to understand both internal controls and ecosystem fluctuations that regulate production of commensal genotoxins is at an early stage. However, as prefaced in the section above, studies are beginning to reveal important environmental cues controlling the size of genotoxic populations or production *per se*. A very recent review summarizing the current understanding of colibactin biosynthesis and its regulation has been published.^111^ For an in-depth discussion of colibactin regulation, please see this excellent synopsis by Addington and colleagues.^111^

**Figure 4.**
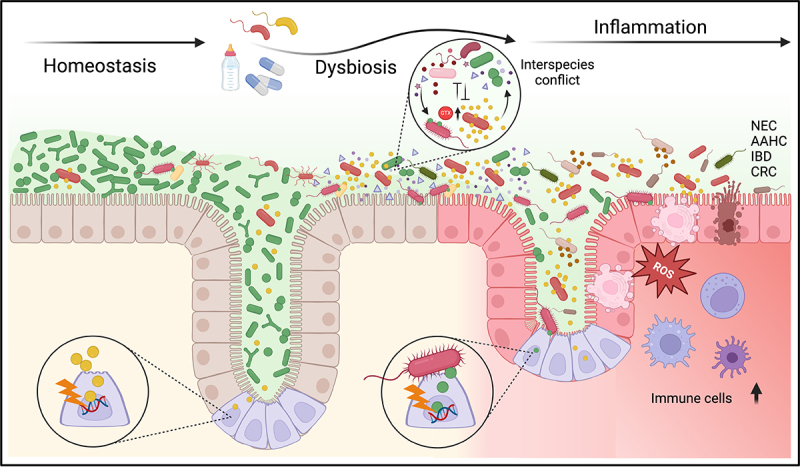
Factors controlling fluctuations in gut intoxication at different life stages remain largely unknown. The initial extra-uterine microbial load originates from the mother and birth environment, then evolves toward a complex consortium. Priority effects, nutrient availability, environmental exposures to novel organisms and interspecies conflict can shift the maternal lineage and influence genotoxin production. Multiple factors including dietary practice, medication and infection further impact the assemblage. In some cases, the resulting dysbiotic state – hallmarked by reduced diversity and radical shifts in relative abundance – drives expansion of genotoxic species and can alter genotoxin production. Intoxication of the gut ecosystem contributes to the etiology of intestinal diseases including colitis (NEC, AAHC), IBD, and CRC. Both intermittent and chronic inflammation expands the aerobic niche while increasing disease risk and severity. Concomitantly the hyper-mutational environment alters microbiome composition and function, enriches the antibiotic resistance reservoir and incites interspecies conflict. Critical interactions with other microbes may influence genotoxin production and effect disease development. Created in BioRender. Kienesberger, S. (2024) BioRender.com/t07l926.

The neonate gut can be seeded by genotoxic commensal organisms (Figure 1) from maternal (birth canal, feces, skin, breast milk) and environmental sources (hospital setting, home environment, other humans). Successful colonization and persistence are shaped by nutrition, host immunity and drug exposure (Figure 4).^112,113^ Conditions favoring the growth of opportunistic pathogens including *K. oxytoca* in the infant gut include a diet of formula milk compared to exclusively human milk.^114,115^ Other factors driving dysbiosis – including antibiotic treatment, and chemical- and pathogen-induced intestinal inflammation – generally result in the loss of microbial density and diversity allowing commensal enterobacteria to bloom relative to strict anaerobes.^116–118^ Inflammation-induced dysbiosis is important for the outgrowth of *E. coli*, but this may vary depending on the intestinal environment.^119,120^ Inflammation, and the accompanying limitation of iron availability were also proposed to upregulate the expression of *pks* genes.^121^

Overgrowth of β-lactamase-producing *Klebsiella* populations is a frequent off-target effect of antibiotic use. Exposure in newborns increases the risk of developing NEC.^122,123^ The metagenomic analysis of fecal samples from premature infants by Olm et al.^45^ found that *Klebsiella* and increased rates of replication in gut bacteria, particularly the *Enterobacteriaceae*, preceded the onset of NEC. Similarly, the facultative anaerobic enteric bacterium *M. morganii* has intrinsic resistance to multiple antibiotics^52^ and antenatal exposure to ampicillin/amoxicillin in premature infants is a factor in *M. morganii*-associated neonatal sepsis. *M. morganii* also shows resistance to gentamycin. Population dynamics of *M. morganii* are thus particularly influenced during childhood when ampicillin and gentamycin are commonly administered. Since mature gut microbiota also respond sensitively to diet and drug intake as well as the onset of a disease or infection, flares of genotoxin exposure are possible across the lifespan. For example, fluctuating selection due to antibiotic therapy is also an etiologic factor driving high intraluminal concentrations of the *til* metabolites and colitis in older children and adults during AAHC.^4,72,124^

## The role of competition sensing and interspecies signaling

During the initial assembly of biota priority effects vary the outcomes of species interactions according to the order of arrival and/or the abundances of resident species and thus shape microbiota assembly and stability.^125^ Current knowledge of priority effects describes mainly ecological interactions that affect resource availability or offer protection from chemical or physiological stress.^126^ Recent studies make a compelling case that mechanisms of interbacterial antagonism also have a significant impact during colonization of the neonatal gut.^19,22,127,128^ Genotoxic strains have the potential to affect maternal lineages and their successors not only by resource competition but by elevating mutation rates. On the one hand, genetic variation can confer advantages to organisms colonizing naïve habitats,^129^ but increasing mutational burden will ultimately exert bactericidal effects.

Studies of other forms of bacterial antagonism suggest that there are large tradeoffs between deploying aggressive phenotypes and investing resources in growth.^19^ Consistent with this view, commensal genotoxicity has been linked to nutrient availability and interspecies signaling. Production of the colibactin prodrug scaffold in *pks+ E. coli* can be substantially reduced *in vitro* when monoculture is replaced by co-cultivation with murine fecal bacteria.^76^ Conversely, inflammation and metabolic competition over trace minerals stimulates colibactin production.^111,119,120,130^ In mixed species communities containing a *Staphylococcus aureus* competitor, Wong et al.^89^ determined that a two component signaling system of *E. coli*, BarA-UvrY, processes environmental information to upregulate toxicity. This mechanism relies on the carbon storage global regulatory system, and thus links primary metabolism to virulence-associated traits.^89^ *til* gene expression is modulated by carbohydrate availability and uptake, and the leucine-responsive regulatory protein Lrp.^71,97,109,131,132^ Osbelt´s study of *K. oxytoca’s* competitive interactions with the enteric pathogen *S*. Typhimurium highlights the importance of nutrient availability.^97^ *til+ Klebsiella* dedicated resources to tilimycin-mediated killing when simple carbohydrates were abundant, while genotoxin-independent activities predominated when carbohydrates were scarce. Moreover, by comparing quantities of *til* metabolites in different experimental intestinal environments this study revealed that factors other than nutrient limitation may alter genotoxin production. Introduction of *til+ Klebsiella* alone to germ-free mice supported abundant secretion of genotoxin indicating that production *in vivo* does not depend on cues from competing bacteria. Nonetheless, genotoxicity did vary according to community composition. *K. oxytoca* secreted the highest levels of tilimycin and mounted the most effective defense against *S*. Typhimurium in a specific pathogen-free (SPF) microbiota disrupted by antibiotic treatment. Notably, pathogen invasion of the gut in this model incited higher levels of tilimycin production by the resident *Klebsiella* than in the pre-infection state; thus *til*+ bacteria can apparently upregulate genotoxicity in response to interspecies exchange. Conversely, a twelve-member bacterial consortium that altered the carbon sources available *in vivo* from those present in the SPF model limited both *Klebsiella* expansion and *til* metabolite secretion in mice. The question of whether members of this community actively antagonize tilimycin production is yet to be investigated.

These early observations are intriguing because to date we have very limited knowledge of whether and how interspecies conflict or even reciprocal antagonism may stimulate genotoxicity in the human host (Figure 3). Bacteria sense danger signals and noxious secretions released by other microbes by various mechanisms. The competition sensing hypothesis predicts that when bacteria sense harm, they will mount an aggressive offense.^133^ Bacterial stress responses to cellular damage by microbial effectors can upregulate not only an appropriate repair system but also their own antagonistic mechanisms. In this context, DNA stress unleashed in the broader community foreshadows a role for commensal genotoxins in a general escalation of competitive interference. In different laboratory settings, bacteria exposed to colibactin or tilimycin initiate a DNA damage response and colibactin (but not tilimycin) increases intracellular accumulation of reactive oxygen species.^58,71^ *In vitro* cultivation of *pks*+ bacteria in mixed communities did not lead to upregulation of colibactin production, but conversely, exposure to colibactin was shown to incite Shiga toxin production by *Citrobacter rodentium* harboring *stx*_*2dact*_ prophage.^59^ That outcome illustrates the potential for toxin-based modulation of bacterial behaviors to impact host health indirectly.

Emerging evidence suggests that bacterial genotoxicity or the retaliation it provokes has ecological impact during early human gut community development (Figure 4). Traces of *til* metabolites have been detected in feces of healthy infants within the first weeks of life.^50^ Mäklin et al.^134^ analyzed fecal metagenomes of infants^42^ and identified significant antagonistic relationships between *E. coli* and *K. grimontii, K. michiganensis*, and *K. pneumoniae*, and similarly between *E. coli* and *E. faecalis*. The Matson laboratory monitored the dynamics of microbiota assembly in the intestines of preterm infants.^74^ To supersede genus level resolution of the *Klebsiella* Coleman, et al.^74^ used a novel deep-sequencing approach to generate amplicon sequence variant (ASV) fingerprints to discriminate between the KoSC and the *K. pneumoniae* species complex (KpSC).^74^ They found that *Klebsiella* species colonized most preterm infants, were more prevalent in NEC subjects versus controls, and replaced *Escherichia* in NEC subjects. Notably, they also found that after initial colonization by multiple distinct strains, over time single KoSC or KpSC ASV fingerprinted strains dominated the gut microbiota, presumably due to interspecies *Klebsiella* competition. Moreover, *Enterococcus faecalis* was frequently co-dominant with KoSC but not with KpSC. Studies in mice^94,96^ have shown that KoSC members *K. pasteurii* and *K. oxytoca* compete very effectively against *K. pneumoniae* in resource competition. Mouse models were also used to demonstrate that the antibacterial and pro-mutagenic activities of tilimycin add a substantial competitive advantage to KoSC over *K. pneumoniae* in the intestine.^71^ In these communities, tilimycin also enhanced *Enterococcus* growth and restricted *E. coli* colonization consistent with observations in infant cohorts.^74^ In the patient groups of premature infants studied by Coleman, tilimycin-producing KoSC members were identified in most NEC subjects and were less frequent in controls. In other infant cohorts, secondary metabolite gene clusters dedicated to quorum sensing and antimicrobial peptide production were associated with NEC onset.^45^ Therefore, the questions of whether and how interspecies conflict can increase the expression of virulence factors in human neonates, specifically in the context of NEC warrants further investigation.

## Outlook

It is becoming increasingly clear that the gut ecosystem represents a rich reservoir of diverse and potentially abundant natural products that target DNA. Discovery of additional members of the bacterial genotoxin family will be a focus of further investigation. The generalizable approach described by Elmassry et al.^55^ – combining quantitative metagenomic analysis, synthetic biology, and murine disease models – represents a powerful tool for discovering molecular mediators of microbiome-associated diseases. Reductionist models of intestinal environments will continue to play an essential role in the detailed characterization of these effectors but developing approaches to interrogate the metabolic and environmental cues that modulate genotoxin production in rich, complex microbiota remains a major challenge. Moreover, we need to understand how interspecies conflict incites genotoxicity and identify defensive mechanisms evolved by the microbiota which may have translational potential. In this context, longitudinal fecal metagenomic and metabolomic analyses of NEC infants and healthy controls harboring toxigenic *Klebsiella* is a powerful approach for discovering community structures and dynamics that either favor or repress genotoxicity.

Greater understanding of these contextual factors may ultimately offer critical insights for targeted therapeutics or inform broader intervention strategies to mitigate ecosystem disruption and genotoxin-mediated diseases. Bearing in mind that commensal genotoxins are deployed to harm competing bacteria can provide valuable insights. Here, we note one salient example. Booth et al.^135^ studied two major categories of bacterial weapons: short-range, contact-dependent toxins and long-range, diffusible ones. They found that diffusible toxins are most effective against susceptible bacterial cells when producer cell frequency and density is high; suggesting successful intervention strategies will need to limit population expansion. By contrast, injected toxins are generally effective regardless of whether a strain is rare or abundant, thus concentrating on colibactin production may be most prudent. Indeed, ongoing efforts explore inhibition through anti-inflammatory medication or dietary interventions including possibly nutraceuticals, prebiotics and probiotic-based functional foods.^111,121,136^ Others aim to subvert contact dependence and limit adherence of *pks+* bacteria to host epithelial cells. This can include medications designed to mitigate dysfunctional mucus structures in vulnerable patient groups^137^ or probiotic consumption. The products of probiotic metabolism are defined as postbiotics and include bioactive compounds such as short-chain fatty acids (SCFAs), peptides, enzymes, and various metabolites. They modulate immune responses, strengthen the intestinal epithelial barrier, and have anti-inflammatory and antioxidant effects.^138^ High concentrations of SCFAs in the lower intestinal tract promote an acidic intracellular environment that inhibits replication of the Enterobacteriaceae.^139,140^ Thus, the fermentation pathways used by probiotic strains are predicted to curtail the expansion of both *pks*+ bacteria as well as producers of diffusible genotoxins such as tilimycin. Consistent with this notion, oral administration of *Bifidobacterium* and *Lactobacillus* modifies the preterm infant gut environment with increased acetate and lactate levels as well as lower fecal pH.^141^ Concurrent reduction in the relative abundance of *Klebsiella*, *Escherichia* and *Enterobacter* was observed. As overgrowth of *til+ Klebsiella* is frequently fueled by antibiotic therapies at many life stages, patients might benefit from concomitant interventions with probiotics to rescue total SCFA production as well as colonic acidification. Notably, however, in the gut ecosystem (i) introduced organisms will undoubtedly encounter antagonism, and (ii) given that commensal genotoxins drive bacterial evolution, protective measures may need to consider the impact of evolutionary dynamics. Alternatively, postbiotics may be an attractive choice as these are characterized by non-viable microbial cells. Finally, chemical modulation of gut microbial functions is another approach toward therapeutic intervention that may overcome some of these challenges and facilitate basic research into the mechanisms of microbiota-host interactions.^76,142^
